# Incidence and characteristics of Lyme neuroborreliosis in adult patients with facial palsy in an endemic area in the Netherlands

**DOI:** 10.1017/S0950268819000438

**Published:** 2019-03-21

**Authors:** S.M. Bierman, B. van Kooten, Y.M. Vermeeren, T.D. Bruintjes, B.C. van Hees, R.A. Bruinsma, G.W. Landman, T. van Bemmel, T.P. Zomer

**Affiliations:** 1 Lyme Centre Apeldoorn, Gelre Hospital, Apeldoorn, The Netherlands; 2 Department of Neurology, Gelre Hospital, Apeldoorn, The Netherlands; 3 Department of Internal Medicine, Gelre Hospital, Apeldoorn, The Netherlands; 4 Department of Otorhinolaryngology, Gelre Hospital, Apeldoorn, The Netherlands; 5 Department of Medical Microbiology and Infection Prevention, Gelre Hospitals, Apeldoorn and Zutphen, The Netherlands; 6 Department of Pediatrics, Gelre Hospital, Apeldoorn, The Netherlands

**Keywords:** Borrelia, facial palsy, idiopathic facial palsy, Lyme neuroborreliosis, tick-borne disease

## Abstract

Making a distinction between facial palsy due to Lyme neuroborreliosis (LNB) and idiopathic facial palsy (IFP) is of importance to ensure timely and adequate treatment. The study objective was to assess incidence and patient characteristics of facial palsy due to LNB. Hospital records were reviewed of adult patients with facial palsy visiting the departments of neurology and/or otorhinolaryngology of Gelre hospitals between June 2007 and December 2017. Gelre hospitals are located in an area endemic for Lyme borreliosis. Patients with LNB had pleocytosis and intrathecal antibody production or pleocytosis with positive IgG serology. Patients with IFP had negative serology. Clinical characteristics were compared between patients with LNB and patients with IFP. Five hundred and fifty-nine patients presented with facial palsy, 4.7% (26) had LNB and 39.4% (220) IFP. The incidence of facial palsy due to LNB was 0.9/100 000 inhabitants/year. Over 70% of patients with facial palsy due to LNB did not report a recent tick bite and/or erythema migrans (EM). Patients with facial palsy due to LNB presented more often in July to September (69.2% *vs.* 21.9%, *P* < 0.001), and had more often headache (42.3% *vs.* 15.5%, *P* < 0.01). To reduce the risk of underdiagnosing LNB in an endemic area, we recommend testing for LNB in patients with facial palsy in summer months especially when presenting with headache, irrespective of a recent tick bite and/or EM.

## Introduction

Lyme borreliosis (LB) is caused by *Borrelia (B.) burgdorferi* sensu lato. It is a tick-borne disease that is transmitted by ticks of the genus Ixodes. In Europe, the spirochetes *B. afzelii*, *B. garinii*, *B. burgdorferi* sensu stricto, *B. spielmanii* and *B. bavariensis* are the most important species to cause LB [1]. In North America only *B. burgdorferi* sensu stricto is known to cause LB. The difference in *Borrelia* species between Europe and North America results in different clinical manifestations of LB. Borrelial lymphocytoma and acrodermatitis chronica atrophicans are common in Europe, but very rarely occur in North America [2]. In addition, Lyme arthritis more often occurs in North America, while Lyme neuroborreliosis (LNB) is more common in Europe [2]. Both in Europe and North America, the most common manifestation of LB is the skin lesion known as an erythema migrans (EM).

LNB is an acute disease which develops within weeks after a bite of an infected tick [2]. Manifestations of LNB are meningo-radiculitis, meningitis and peripheral facial palsy [1]. Other causes of peripheral facial palsy are autoimmune diseases, trauma, tumours, metabolic diseases and a variety of infectious diseases, including herpes simplex infection and varicella zoster infection [3]. However, up to 80% of adult patients present with idiopathic facial palsy (IFP) [3, 4]. IFP is a diagnosis reached by a process of exclusion of other reasonable diagnoses [5].

In the Netherlands, patients with facial palsy generally first contact their general practitioner, who can refer to either a neurologist and/or an otorhinolaryngologist. The Dutch guideline on LB recommends serologic testing for LB if patients present with bilateral facial palsy or unilateral facial palsy with other signs of possible LB, such as a recent EM and/or a tick bite [6]. Serology should consist of two-tier testing in which a positive or indeterminate immunoglobulin M (IgM) and immunoglobulin G (IgG) enzyme-linked immunosorbent assay (ELISA) is confirmed with an immunoblot. When serology for LB is positive or when serology is negative with a strong clinical suspicion for LNB, a lumbar puncture for testing for pleocytosis and intrathecal antibody production is warranted. When serology is negative without a strong clinical suspicion for LNB, serological testing should be repeated 2–4 weeks later when symptom duration is <8 weeks. Facial palsy can be considered to be caused by LNB if there is no other obvious reason, pleocytosis and intrathecal IgM and/or IgG Borrelia-specific antibody production, or if there is pleocytosis without intrathecal antibody production, but with positive IgG serology [7].

The guidelines recommend that patients with facial palsy due to LNB are treated with a third-generation cephalosporin antibiotic ceftriaxone, whereas treatment for IFP includes prednisone [5, 6]. Treatment of LNB with both prednisone and antibiotics has been associated with worse long-term facial function outcome compared to treatment with solely antibiotics [8]. Therefore, in order to treat a patient adequately it is necessary to timely diagnose the cause of the facial palsy. Timely initiation of treatment for LNB has been associated with fewer residual symptoms [9]. However, this is hampered as the sensitivity of tests for *Borrelia* antibodies in serum and cerebrospinal fluid (CSF) is limited in the early phase of LNB [10, 11].

Certain patient characteristics might facilitate timely diagnosis of facial palsy due to LNB. Few previous studies have assessed differences in accompanying symptoms of LNB patients and patients with IFP. These studies reported that onset in summer season, enlarged cervical lymph nodes, arthralgia and neurological symptoms were more common in patients with LNB [12, 13]. More studies on this are needed as these studies included small number of patients with facial palsy due to LNB. Moreover, no studies on the incidence and patient characteristics of patients with facial palsy due to LNB have been performed in the Netherlands. The study objective was to assess incidence of facial palsy due to LNB and patient characteristics to discriminate between patients with facial palsy due to LNB and IFP in clinical practice.

## Methods

### Study population

The study population consisted of adult patients with facial palsy visiting the departments of neurology and/or otorhinolaryngology of Gelre hospitals in Apeldoorn and Zutphen in the Netherlands between June 2007 and December 2017. Gelre hospitals are located in an endemic area for LB [14]. Based on five diagnosis treatment combination codes (i.e. for the department of otorhinolaryngology: abnormalities of the nervus facialis, and for the department of neurology: N. VII, specific neuro-infections, eye movement disorders (N. III/IV/VI) and other brain nerves) a list of possible patients with facial palsy was obtained. Patients were excluded when there was no evident facial palsy or when the medical consultation concerned a follow-up visit of a previous facial palsy outside the study period.

### Data collection

Hospital records were reviewed for the following data: date of first medical consultation for evaluation of the facial palsy, diagnosis, bilateral or unilateral (left or right-sided) facial palsy, recent tick bite and/or EM, symptoms (i.e. headache, watery eye, eye closure problems, earache and taste dysfunction), treatment, number of follow-up visits and recovery. Complete recovery was defined as a House–Brackmann (HB) score of I or when complete recovery of the facial palsy was recorded in the hospital record. The HB score at first medical consultation and last follow-up was recorded. HB scores are widely used to characterise the degree of facial palsy and range from grade I representing normal facial function to grade VI representing complete paralysis [5]. In addition, laboratory data were collected including results of serological tests, leucocyte cell count in CSF and intrathecal *Borrelia* antibody production. When data on symptoms, a tick bite or EM were missing in the hospital record, these variables were considered not present. Patients for which data on recovery were missing were excluded from those analyses. Data were systematically recorded using Microsoft Access. After data entry, 30% of the records were double checked.

### Guideline compliance

Descriptive analyses were conducted to assess how often LB is addressed in anamnesis when patients present with facial palsy, how often *Borrelia* serology was tested and when negative retested, and how often a lumbar puncture was performed. LB anamnesis was positive when patients reported a recent tick bite and/or EM, or activities with possible tick exposure, such as, e.g. hiking in the forest. Anamnesis was negative when patients reported this was not the case, or LB in anamnesis was not recorded.

### Patient classification

Patients were classified as having facial palsy due to LNB or IFP. LNB was defined as having a facial palsy without other detected cause, pleocytosis and intrathecal IgM and/or IgG *Borrelia*-specific antibody production. In case of pleocytosis and no intrathecal antibody production, *Borrelia* IgG serology had to be positive for classification as LNB [7]. IFP was defined as a facial palsy without a known cause in combination with negative IgM and IgG *Borrelia* serology. If hospital records stated the facial palsy as idiopathic or Bell's palsy, or no other cause was recorded, the researchers concluded that the facial palsy was idiopathic.

### Laboratory data

Serological tests for *B. burgdorferi* consisted of an IgM and IgG ELISA, followed by an immunoblot. In case only an ELISA was performed and no immunoblot, the ELISA result was leading. If both an ELISA and immunoblot were performed, the immunoblot result was leading [6]. An indeterminate ELISA without an immunoblot was considered negative, as well as an indeterminate immunoblot. The ELISAs used were the Enzygnost Lyme Link VlsE/IgG and the Enzygnost Borreliosis IgM by DADE Behring, Marburg, Germany [15]. The immunoblot used was the *recom*Line *Borrelia* IgG and IgM immunoblot of Mikrogen, Neuried, Germany [16]. Pleocytosis was defined as leucocyte cell count in CSF >5 per μl or >15 per 3 µl [17]. Intrathecal *Borrelia* antibody production was detected by analysing a serum and CSF pair with the IDEIA Lyme neuroborreliosis OXOID, Hampshire, UK [18]. Diagnostic tests were performed as indicated by the manufacturers.

### Statistics

Overall incidence of facial palsy and the incidence of facial palsy due to LNB was calculated per 100 000 inhabitants per year with 95% confidence interval (CI). To calculate the incidence, the service area of Gelre hospitals in Apeldoorn and Zutphen was used, which included approximately 280 000 inhabitants per year during the study period. Seasonal variation was assessed with the percentage of patients per month.

Patients with a facial palsy due to another cause than LNB were described and excluded from further analyses. Excluded from further analyses were also patients that had IFP in which *Borrelia* serology was not tested, as there was nothing known about these patients in relation to potential LB. Patients with positive *Borrelia* serology that did not have pleocytosis and intrathecal IgM and/or IgG *Borrelia*-specific antibody production were also excluded, as these patients did not have LNB, but possibly other (previous) manifestations of LB.

Overall frequencies and frequencies specific for patients with LNB and IFP were calculated and compared using the *χ*^2^ and Fisher's exact tests. Medians were compared with the Mann–Whitney *U* test. *P*-values <0.05 were considered significant. Statistical analyses were performed using SPSS version 25 (SPSS Inc, Chicago, Illinois, USA).

As data were collected as part of usual care, the study falls outside the scope of the Dutch Medical Research Involving Human Subjects Act and no informed consent was required. This was confirmed by the Medical Ethics Committee of Isala hospital Zwolle.

## Results

In total, 3507 records were reviewed. Five hundred and fifty-nine patients presented with facial palsy with a median age of 53 years (range 18–89) and 52.6% (294) were male. The overall incidence of facial palsy was 19.0 (95% CI 17.5–20.6) per 100 000 inhabitants per year.

Of 559 patients with facial palsy, 4.7% (26) were recorded with LNB, 39.4% (220) with IFP and negative *Borrelia* serology, 30.4% (170) with IFP and no *Borrelia* serology tested, and 7.7% (43) did not have LNB but positive *Borrelia* serology (i.e. 16 IgM ELISA, seven IgG ELISA, two both IgM and IgG ELISA, seven IgM immunoblot, eight IgG immunoblot, three both IgM and IgG immunoblot). In addition, 17.9% (100) of patients were recorded with a facial palsy due to another cause than LB of which 67.0% (67) concerned other infectious diseases, 11.0% (11) tumours, 10.0% (10) traumas, 6.0% (six) auto-immune disorders and 6.0% (six) other causes.

**Fig. 1.**
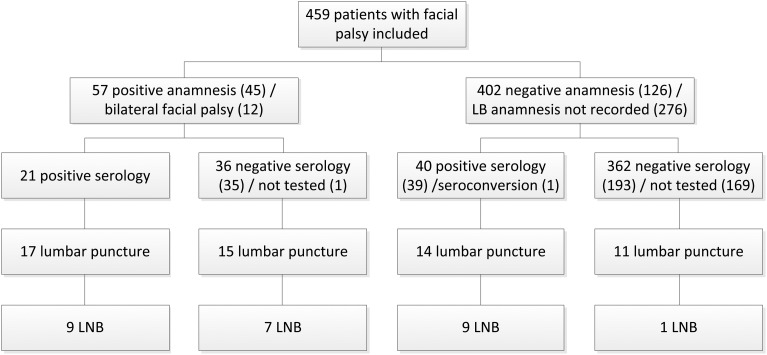
Flow chart of 26 patients with Lyme neuroborreliosis who presented with facial palsy at the departments of neurology and otorhinolaryngology of Gelre hospitals between June 2007 and December 2017. LB, Lyme borreliosis; LNB, Lyme neuroborreliosis.

There were 459 patients with an unknown cause of the facial palsy. Figure 1 shows the number of these patients with LB addressed in anamnesis, serology tested and lumbar punctures performed. In 38.5% (10) of 26 LNB patients, the anamnesis was negative for LB or nothing was recorded concerning LB in anamnesis. There were eight patients with LNB and negative serology, of which one patient also had a negative anamnesis. A lumbar puncture was performed in 57 patients of which 25 had a negative anamnesis or nothing was recorded concerning LB in anamnesis. Of 205 patients with negative serology and no lumbar puncture to test for LNB, 15.1% (31) patients were retested several weeks later (median 6 weeks, range 0–34 weeks). Of 31 patients, one patient seroconverted from a negative to positive IgG immunoblot. After seroconversion, a lumbar puncture was performed and LNB was confirmed.

Of 26 patients with LNB, 23 had pleocytosis and intrathecal *Borrelia*-specific antibody production and three had pleocytosis with positive IgG serology. The median number of leucocytes in CSF was 602 (range 21–1952). Intrathecal *Borrelia*-specific antibody production of both IgM and IgG was found in 53.8% (14) of the patients (Table 1). The incidence of facial palsy due to LNB was 0.9 (95% CI 0.6–1.3) per 100 000 inhabitants per year. Four of 26 patients with facial palsy due to LNB also reported radiculopathy.

**Table 1.** Laboratory characteristics of 26 patients with facial palsy due to Lyme neuroborreliosis presenting between June 2007 and December 2017 at Gelre hospitals.

Laboratory characteristics	% (*n*) *N* = 26
Serology results	
IgM ELISA-positive	11.5 (3)
IgG ELISA-positive	3.8 (1)
IgM and IgG ELISA-positive	30.8 (8)
IgG immunoblot-positive	19.2 (5)
IgM and IgG immunoblot-positive	11.5 (3)
IgM and IgG immunoblot-negative	23.1 (6)
Intrathecal antibody production	
IgM-positive	11.5 (3)
IgG-positive	23.1 (6)
IgM and IgG-positive	53.8 (14)
IgM and IgG-negative^a^	11.5 (3)

a Three patients with no intrathecal antibody production had a positive IgM and IgG ELISA.

**Fig. 2.**
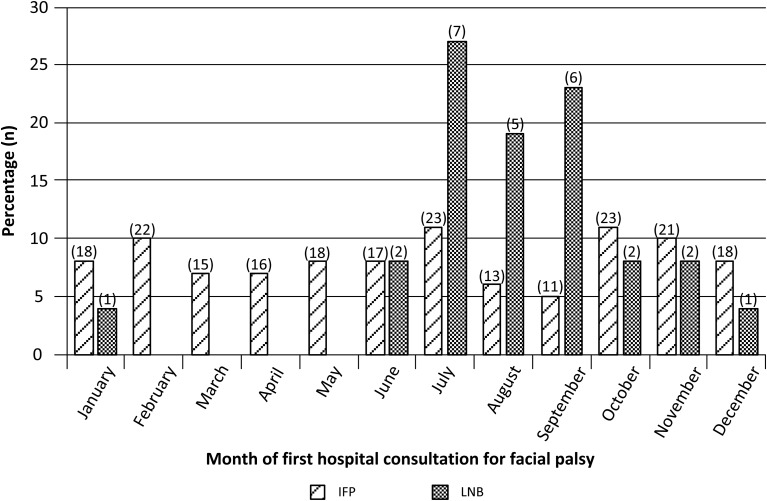
Percentage (number) of patients per month with facial palsy due to Lyme neuroborreliosis (LNB) (*n* = 26) and idiopathic facial palsy (IFP) (*n* = 215). Data of five patients with IFP were missing.

Patients with LNB presented from June until January with a peak in July, August and September (Fig. 2). Patients with IFP presented throughout the whole year ranging from 5% to 11% per month. In summer (July, August, September), 69.2% (18/26) of the patients with LNB presented *vs.* 21.9% (47/215) of patients with IFP (*P* < 0.001). Patient characteristics that occurred more frequently among patients with LNB than among patients with IFP were bilateral facial palsy (19.2% *vs.* 2.7%, respectively; *P* < 0.01), a recent tick bite and/or EM (26.9% *vs.* 4.1%, respectively; *P* < 0.001) and headache (42.3% *vs.* 15.5%, respectively; *P* < 0.01) (Table 2). Of seven patients with LNB, five had a recent tick bite, one a recent EM and one patient reported both a recent tick bite and recent EM.

**Table 2.** Comparison of characteristics of patients with facial palsy due to Lyme neuroborreliosis (LNB) and idiopathic facial palsy (IFP) presenting in Gelre hospitals between June 2007 and December 2017

	Total % (*n*) (*N* = 246)	LNB % (*n*) (*N* = 26)	IFP % (*n*) (*N* = 220)	*P*-value
Clinical data at first medical consultation				
Median age (range)	50 (18–88)	49 (19–78)	51 (18–88)	0.321
Gender (male)	54.5 (134)	61.5 (16)	53.6 (118)	0.444
Bilateral facial palsy	4.5 (11)	19.2 (5)	2.7 (6)	0.003
Unilateral facial palsy right-sided	47.4 (110/232)	33.3 (7/21)	48.8 (103/211)	0.175
Median HB score (range)	IV (I–VI) (*n* = 162)	III (II–VI) (*n* = 8)	IV (I–VI) (*n* = 154)	0.239
Recent tick bite and/or EM	6.5 (16)	26.9 (7)	4.1 (9)	<0.001
Activities with tick exposure	17.9 (44)	61.5 (16)	12.7 (28)	<0.001
Symptoms				
Headache	18.3 (45)	42.3 (11)	15.5 (34)	0.002
Watery eye	11.8 (29)	7.7 (2)	12.3 (27)	0.749
Eye closure problems	28.9 (71)	42.3 (11)	27.3 (60)	0.110
Earache	18.3 (45)	19.2 (5)	18.2 (40)	1.000
Taste dysfunction	13.4 (33)	11.5 (3)	13.6 (30)	1.000
Timeliness				
Median number of days between onset of facial palsy and first hospital contact (range)	4 (0–272)^a^ (*n* = 240)	4 (0–21) (*n* = 26)	3.5 (0–272) (*n* = 214)	0.828
Median number of days between onset of facial palsy and last follow-up visit (range)	66 (3–536) (*n* = 221)	113 (35–529) (*n* = 25)	59 (3–536) (*n* = 196)	0.002
Median number of days between first hospital contact and last follow-up visit (range)	56.5 (2–532) (*n* = 224)	110 (31–528) (*n* = 25)	52 (2–532) (*n* = 199)	<0.001
Recovery				
Median number of follow-up visits (range)	2 (0–12) (*n* = 240)	3 (0–7) (*n* = 26)	2 (0–12) (*n* = 214)	0.157
Median HB score at last follow-up visit (range)	II (I-IV) (*n* = 33)	III (*n* = 1)	II (I-IV) (*n* = 32)	0.209
Recovery within 6 months after first hospital contact	78.4 (134/171)	82.4 (14/17)	77.9 (120/154)	1.000
Recovery within 18 months after first hospital contact	77.7 (160/206)	82.6 (19/23)	77.0 (141/183)	0.546

EM, erythema migrans; HB score, House–Brackmann score.

a For four patients, the duration between start of facial palsy and first hospital contact was more than 6 weeks.

Concerning treatment, among 220 patients with IFP, 72.7% (160) received prednisone. Among 26 patients with LNB, 50.0% (13) received prednisone. All 26 patients with LNB received ceftriaxone (one patient received doxycycline followed by ceftriaxon). For these 26 patients, the median number of days between first hospital contact and start of antibiotic treatment was 1 (range 0–257). Among patients with IFP, three patients were treated with ceftriaxone, three with doxycycline and one with amoxicillin. The number of days between first hospital contact and last follow-up visit was significantly longer for patients with facial palsy due to LNB compared to patients with IFP (Table 2). Regarding recovery, there was no significant difference in the number of follow-up visits, HB score or proportion of patients with recovery when comparing patients with facial palsy due to LNB and patients with IFP (Table 2).

## Discussion

This study found that the incidence of facial palsy due to LNB was low in an area endemic for LB in the Netherlands. Of patients referred to a hospital with facial palsy 5% had LNB. That over 70% of patients with facial palsy due to LNB did not report a recent tick bite and/or EM shows patients might benefit from serologic testing and a lumbar puncture to rule out LNB irrespective of whether they had a recent tick bite and/or EM. Patient characteristics associated with LNB were presentation in summer months and headache. To reduce the risk of underdiagnosing LNB in an endemic area, we recommend testing for LNB in patients with facial palsy in summer especially when presenting with headache, irrespective of a recent tick bite and/or EM.

Five per cent of patients with facial palsy had LNB, which is in line with previous studies in which 2 to 16% of patients with facial palsy had LNB [12, 19–22]. In studies including only children, this prevalence ranged from 34 to 65% [17, 23–25]. The incidence of facial palsy due to LNB was 0.9 patients per 100 000 inhabitants per year. In 2010, the incidence of LNB in the Netherlands was 2.6 (95% CI 2.4–2.8) per 100 000 inhabitants [26]. The incidence of LNB in our study was lower as facial palsy is only one of several manifestations of LNB. A Dutch study in 2001 reported that 40% (8/20) of LNB patients had facial palsy [27]. This percentage is comparable to results from a Danish study in which 38.7% of adult patients with LNB had facial palsy [9].

The number of patients with LNB in this study may be an underestimation. Possibly patients with facial palsy presented at other departments than neurology or otorhinolaryngology and are therefore not included in this study. Sensitivity of serological tests in early LB is limited and false-negative serology results occur [10, 11]. Therefore, guidelines state that patients suspected of LB with negative *Borrelia* serology should be retested two to four weeks later when duration of symptoms is less than eight weeks [6]. This study demonstrated that the number of patients retested is low. Only 31 patients with negative serology and no lumbar puncture were retested and in one patient LNB was diagnosed following seroconversion. There is a need to raise awareness among neurologists and otorhinolaryngologists to retest in order to minimise underdiagnosing LB. In this study, there were eight patients with negative serology who had LNB with pleocytosis and intrathecal antibody production in CSF. This demonstrates the need to lower the threshold for lumbar punctures to rule out LNB. As the number of lumbar punctures was low in this study, LNB patients may be missed. Timeliness of diagnosis can be improved by performing lumbar punctures instead of retesting several weeks later.

This study showed a seasonal distribution of patients with facial palsy due to LNB with a peak in July, August and September. This has been reported in previous studies [20, 28]. In summer months, both tick activity and exposition to ticks increase due to the warmer weather. Other characteristics associated with LNB, like bilateral facial palsy, or a tick bite and/or EM, have been reported before and are addressed in guidelines [5, 6]. It is noteworthy that over 70% of patients with LNB did not have an EM and/or tick bite. Therefore, the absence of an EM or tick bite does not exclude LNB [13].

A Finnish prospective study among 503 patients with facial palsy of which 11 had LNB also found an association between headache and LNB [12]. In this Finnish study, both adults and children were included. To our knowledge, our study is one of the first studies to find an association between LNB and headache specifically in adults.

In this study, there was no difference in recovery between patients with facial palsy due to LNB or IFP. Recovery was based on the facial nerve function assessed by a medical specialist and did not take into account non-specific long-term symptoms. Comparison with previous studies is hampered due to different definitions of recovery and study populations. A study including both children and adults assessed recovery with a questionnaire and reported slightly better outcome in patients with LNB than in patients with IFP [12].

A limitation of our study is that data were collected retrospectively and we relied on the information recorded in the electronic medical records. Other symptoms possibly associated with LNB, like e.g. radiculopathy, were described too seldom to be able to draw conclusions on. Moreover, the retrospective design allows for misinterpretation of data, which was minimised by double-checking 30% of the electronic medical files. Another limitation is that calculation of the incidence was based on the service area of the hospital, which is an estimation of how many inhabitants use the hospital. Fifteen of 559 patients with facial palsy did not live in the service area and 31 patients lived directly surrounding the service area. Inhabitants of the service area might also visit other hospitals.

Strengths of our study are the long study period of 10.5 years, which minimised bias due to yearly variations. In addition, the comparison group consisted of patients with IFP in which *Borrelia* serology was negative, to minimise misclassification. Another strength of our study was that it focused only on adults, as the proportion of facial palsy due to LNB differs between children and adults [9], and most studies on LNB included both adults and children, or solely children.

In conclusion, this study assessed differences between patients with facial palsy due to LNB and IFP. Over 70% of patients with facial palsy due to LNB did not report a recent tick bite and/or EM. Patients might benefit from serologic testing and a lumbar puncture to rule out LNB irrespective of whether they had a recent tick bite or EM. Patient characteristics associated with LNB were presentation in summer months and headache. To reduce the risk of underdiagnosing LNB in an endemic area, we recommend testing for LNB in patients with facial palsy in summer especially when presenting with headache, irrespective of a recent tick bite and/or EM. Moreover, there is a need to raise awareness among medical specialists *Borrelia* serology should be retested several weeks later when serology is negative and no lumbar puncture is performed. Only 15% of 205 patients eligible for retesting were actually retested. Timeliness of diagnosis could be improved by performing lumbar punctures instead of retesting several weeks later, as there were eight patients with LNB, despite negative serology.

## References

[1] Stanek G (2012) Lyme borreliosis. The Lancet 379, 461–473. 10.1016/S0140-6736(11)60103-7 21903253

[2] Stanek G (2011) Lyme borreliosis: clinical case definitions for diagnosis and management in Europe. Clinical Microbiology and Infection 17, 69–79. 20132258 10.1111/j.1469-0691.2010.03175.x

[3] Bašić-Kes V (2013) Peripheral facial weakness (Bell's palsy). Acta Clinica Croatica 52, 195–202. 24053080

[4] Zandian A (2014) The neurologist's dilemma: a comprehensive clinical review of Bell's palsy, with emphasis on current management trends. Medical Science Monitor 20, 83–90. 24441932 10.12659/MSM.889876 PMC3907546

[5] Dutch Quality Institute for Health Care (CBO) (2009) Dutch national guideline for idiopathic peripheral facial palsy. Available at https://www.nvmka.nl/sites/www.nvmka.nl/files/Richtlijn-Idiopathische-Perifere-Aangezichtsverlamming-IPAV.pdf (Accessed 3 July 2018).

[6] Dutch Quality Institute for Health Care (CBO) (2013) Dutch national guideline for Lyme borreliosis. Available at https://www.rivm.nl/sites/default/files/2018-11/CBO%20richtlijn%20Lymeziekte%20definitief%20juli%202013.pdf (Accessed 3 July 2018).

[7] Mygland Å (2010) EFNS guidelines on the diagnosis and management of European Lyme neuroborreliosis. European Journal of Neurology 17, 8–16. 19930447 10.1111/j.1468-1331.2009.02862.x

[8] Jowett N (2017) Steroid use in Lyme disease-associated facial palsy is associated with worse long-term outcomes. The Laryngoscope 127, 1451–1458. 27598389 10.1002/lary.26273

[9] Knudtzen FC (2017) Characteristics and clinical outcome of Lyme neuroborreliosis in a high endemic area, 1995–2014: a retrospective cohort study in Denmark. Clinical Infectious Diseases 65, 1489–1495. 29048514 10.1093/cid/cix568

[10] Leeflang MMG (2016) The diagnostic accuracy of serological tests for Lyme borreliosis in Europe: a systematic review and meta-analysis. BMC Infectious Diseases 16, 140. 27013465 10.1186/s12879-016-1468-4 PMC4807538

[11] Ljøstad U, Skarpaas T and Mygland Å (2007) Clinical usefulness of intrathecal antibody testing in acute Lyme neuroborreliosis. European Journal of Neurology 14, 873–876. 17662007 10.1111/j.1468-1331.2007.01799.x

[12] Peltomaa M (2002) Lyme borreliosis and facial paralysis – a prospective analysis of risk factors and outcome. American Journal of Otolaryngology – Head and Neck Medicine and Surgery 23, 125–132. 10.1053/ajot.2002.123434 12019479

[13] Bremell D and Hagberg L (2011) Clinical characteristics and cerebrospinal fluid parameters in patients with peripheral facial palsy caused by Lyme neuroborreliosis compared with facial palsy of unknown origin (Bell's palsy). BMC Infectious Diseases 11, 215. 21831262 10.1186/1471-2334-11-215 PMC3176206

[14] Hofhuis A (2016) Decrease in tick bite consultations and stabilization of early Lyme borreliosis in the Netherlands in 2014 after 15 years of continuous increase. BMC Public Health 16, 425. 27216719 10.1186/s12889-016-3105-y PMC4877959

[15] Marangoni A (2008) *Borrelia burgdorferi* VlsE antigen for the serological diagnosis of Lyme borreliosis. European Journal of Clinical Microbiology and Infectious Diseases 27, 349–354. 18197445 10.1007/s10096-007-0445-7

[16] Mikrogen Diagnostik (2013) Mikrogen Diagnostik, recomLine Borrelia, Instructions for use. Available at https://www.mikrogen.de/english/products/product-overview/weitereinfo/borrelia-igg-1.html (Accessed 4 July 2018).

[17] Backman K and Skogman BH (2018) Occurrence of erythema migrans in children with Lyme neuroborreliosis and the association with clinical characteristics and outcome – a prospective cohort study. BMC Pediatrics 18, 189. 29890951 10.1186/s12887-018-1163-2 PMC5996539

[18] OXOID IDEIA Lyme Neuroborreliosis. Available at https://www.thermofisher.com/search/results?persona=DocSupport&refinementSearch=true&query=Manuals+&+Protocols&navId=4294959596&refinementQuery=IDEIA+Lyme+neuroborreliosis (Accessed 4 July 2018).

[19] Ljøstad U (2005) Acute peripheral facial palsy in adults. Journal of Neurology 252, 672–676. 15778908 10.1007/s00415-005-0715-1

[20] Kindler W (2016) Peripheral facial palsy as an initial symptom of Lyme neuroborreliosis in an Austrian endemic area. Wiener Klinische Wochenschrift 128, 837–840. 25576332 10.1007/s00508-014-0685-3

[21] Hohman MH and Hadlock TA (2014) Etiology, diagnosis, and management of facial palsy: 2000 patients at a facial nerve center. The Laryngoscope 124, E283–E293. 24431233 10.1002/lary.24542

[22] Roberg M (1991) Acute peripheral facial palsy: CSF findings and etiology. Acta Neurologica Scandinavica 83, 55–60. 1849336 10.1111/j.1600-0404.1991.tb03959.x

[23] Tveitnes D, Øymar K and Natås O (2007) Acute facial nerve palsy in children: how often is it Lyme borreliosis? Scandinavian Journal of Infectious Diseases 39, 425–431. 17464865 10.1080/00365540601105764

[24] Arnež M and Ružić-Sabljić E (2010) Lyme borreliosis and acute peripheral facial palsy in Slovenian children. Pediatric Infectious Disease Journal 29, 182–184. 19952981 10.1097/INF.0b013e3181bbf28a

[25] Nigrovic LE (2008) Clinical predictors of Lyme disease among children with a peripheral facial palsy at an emergency department in a Lyme disease-endemic area. Pediatrics 122, e1080–e1085. 18931349 10.1542/peds.2008-1273

[26] Hofhuis A (2015) Physician reported incidence of early and late Lyme borreliosis. Parasites and Vectors 8, 161. 25889086 10.1186/s13071-015-0777-6 PMC4363353

[27] Kuiper H (2004) Klinisch spectrum en incidentie van neuroborreliose in Nederland. Nederlands Tijdschrift voor Geneeskunde 3, 148. 15106319

[28] Schwartz AM (2017) Surveillance for Lyme disease – United States, 2008–2015. Morbidity and Mortality Weekly Report. Surveillance Summaries 66, 1–12. 10.15585/mmw.ss6622a1 PMC5829628 29120995

